# Chronic wasting disease prions are not transmissible to transgenic mice overexpressing human prion protein

**DOI:** 10.1099/vir.0.024380-0

**Published:** 2010-10

**Authors:** Malin K. Sandberg, Huda Al-Doujaily, Christina J. Sigurdson, Markus Glatzel, Catherine O'Malley, Caroline Powell, Emmanuel A. Asante, Jacqueline M. Linehan, Sebastian Brandner, Jonathan D. F. Wadsworth, John Collinge

**Affiliations:** 1 MRC Prion Unit and Department of Neurodegenerative Disease, UCL Institute of Neurology, National Hospital for Neurology and Neurosurgery, Queen Square, London WC1N 3BG, UK; 2 Department of Pathology, University of California, San Diego, 9500 Gilman Dr., La Jolla, CA 92093-0612, USA; 3 Institute of Neuropathology, University Medical Center Hamburg-Eppendorf, Martinistrasse 52, D-20246 Hamburg, Germany

## Abstract

Chronic wasting disease (CWD) is a prion disease that affects free-ranging and captive cervids, including mule deer, white-tailed deer, Rocky Mountain elk and moose. CWD-infected cervids have been reported in 14 USA states, two Canadian provinces and in South Korea. The possibility of a zoonotic transmission of CWD prions via diet is of particular concern in North America where hunting of cervids is a popular sport. To investigate the potential public health risks posed by CWD prions, we have investigated whether intracerebral inoculation of brain and spinal cord from CWD-infected mule deer transmits prion infection to transgenic mice overexpressing human prion protein with methionine or valine at polymorphic residue 129. These transgenic mice have been utilized in extensive transmission studies of human and animal prion disease and are susceptible to BSE and vCJD prions, allowing comparison with CWD. Here, we show that these mice proved entirely resistant to infection with mule deer CWD prions arguing that the transmission barrier associated with this prion strain/host combination is greater than that observed with classical BSE prions. However, it is possible that CWD may be caused by multiple prion strains. Further studies will be required to evaluate the transmission properties of distinct cervid prion strains as they are characterized.

## INTRODUCTION

Chronic wasting disease (CWD) is a prion disease affecting free-ranging and captive cervids, including mule deer, white-tailed deer, Rocky Mountain elk and moose (Williams & Young, 1980, 1982; Williams, 2005; Baeten *et al.*, 2007). Like all mammalian prion diseases, which include Creutzfeldt–Jakob disease (CJD), kuru and variant CJD (vCJD) in humans and bovine spongiform encephalopathy (BSE) in cattle, the central event in CWD infection is the post-translational conversion of the host-encoded, cellular prion protein (PrP^C^), to an abnormal isoform, designated PrP^Sc^ (Prusiner, 1998; Collinge & Clarke, 2007). Progressive accumulation of PrP^Sc^ in the central nervous system (Guiroy *et al.*, 1993) is associated with clinical signs of CWD which include weight loss, behavioural changes, excessive salivation, difficulty swallowing, polydipsia, polyuria, and ataxia prior to death (Williams & Young, 1980, 1982; Williams, 2005). International concern over CWD is growing as infected cervids have now been reported in 14 states in North America, two Canadian provinces and in South Korea (Kim *et al.*, 2005; Williams, 2005; Sigurdson & Aguzzi, 2007; Sigurdson, 2008). To date, CWD has not been reported in Europe, although surveillance has been limited.

The prevalence of CWD infection can reach levels of up to 30 % in free-ranging herds in North America and up to 90 % in animals housed in CWD research facilities (Williams, 2005). Infectious prions in the saliva (Mathiason *et al.*, 2006; Haley *et al.*, 2009; Mathiason *et al.*, 2009), urine (Haley *et al.*, 2009) and faeces of CWD-infected animals (Tamguney *et al.*, 2009) may underlie the highly efficient natural transmission of CWD among cervids through environmental contamination (Mathiason *et al.*, 2009). Protease-resistant cervid prion protein has recently been demonstrated in an environmental water sample from a CWD endemic area (Nichols *et al.*, 2009).

Despite efficient horizontal transmission of CWD prions among cervids, to date there is no clear evidence for natural disease transmission to other species. A recent survey for transmissible spongiform encephalopathy in scavengers of white-tailed deer carcasses in a CWD endemic area of Wisconsin found no evidence for cross-species transmission (Jennelle *et al.*, 2009). Nevertheless, the zoonotic transmission of BSE prions (Collinge *et al.*, 1996; Hill *et al.*, 1997; Bruce *et al.*, 1997; Asante *et al.*, 2002; Wadsworth & Collinge, 2007) has dramatically highlighted the potential risk posed to humans from dietary exposure to CWD prions (Belay, 2004; Sigurdson, 2008). Infectious prions are present in the blood (Mathiason *et al.*, 2006), skeletal muscle (Angers *et al.*, 2006) and fat (Race *et al.*, 2009a) of CWD-infected deer and CWD prions have been shown to be experimentally transmissible after oral inoculation of elk and deer and cervid PrP expressing transgenic mice (Hamir *et al.*, 2006; Fox *et al.*, 2006; Trifilo *et al.*, 2007). Consumption of hunted deer and elk is widely practised in North America and a survey conducted by the American Red Cross and other blood banking establishments has reported that ∼40 % of USA blood donors have consumed venison obtained from the wild (Belay *et al.*, 2001). To date, however, epidemiological surveillance has not indicated any link between human disease and CWD exposure (Belay, 2004; Mawhinney *et al.*, 2006; Anderson *et al.*, 2007). However, incubation periods in human prion disease even in the absence of a transmission barrier can exceed 50 years (Collinge *et al.*, 2006, 2008). Accordingly, there has been intense research interest in establishing the host range of CWD prions through experimental transmissions to laboratory animals (Tamguney *et al.*, 2006; Raymond *et al.*, 2007; Sigurdson *et al.*, 2008; Heisey *et al.*, 2010) and through the use of *in vitro* prion amplification systems (Raymond *et al.*, 2000; Kurt *et al.*, 2009).

Concern that CWD prions might be transmissible to humans was heightened in 2005 by the finding that squirrel monkeys can be infected by intracerebral inoculation with CWD mule deer brain homogenate (Marsh *et al.*, 2005). However, a more recent study has shown that cynomolgus macaques (that are evolutionarily closer to humans) differ significantly from squirrel monkeys with respect to their susceptibility to infection with CWD prions, with no evidence for clinical disease in macaques at 70 months post-inoculation (Race *et al.*, 2009b). Crucially however, because prion transmission barriers and prion strains are intimately related by conformational selection (Collinge, 1999; Collinge & Clarke, 2007) the ability of CWD prions to propagate in humans cannot be inferred by studying the interaction of CWD prions with distinct (albeit highly conserved) PrP sequences from other species. To date, two studies have reported that transgenic mice expressing human PrP with methionine at polymorphic residue 129 are resistant to intracerebral challenge with CWD prions. The first of these studies used two lines of transgenic mice expressing human PrP at either one or two times the endogenous level of mouse brain. After inoculation with CWD-infected elk brain homogenate, none of these transgenic mice showed clinical signs of prion disease or detectable accumulation of abnormal PrP by either immunohistochemistry or immunoblotting (Kong *et al.*, 2005). Although these mice are susceptible to infection with atypical BSE prions, their susceptibility to classic BSE prions or vCJD prions has not been reported (Kong *et al.*, 2008). The second study used hemizygous transgenic mice expressing human PrP at two times the endogenous level of murine PrP expression in mouse brain. No evidence of clinical prion disease was observed following intracerebral challenge with CWD-infected elk, mule deer or white-tailed deer brain homogenate; however, importantly subclinical infection was not excluded (Tamguney *et al.*, 2006). Susceptibility of these mice to infection with BSE or vCJD prions has not been reported. Here, to investigate further the potential risks for transmission of cervid prions to humans, we have transmitted mule deer CWD prions to lines of transgenic mice overexpressing human PrP two- to sixfold with either methionine or valine at polymorphic residue 129 in which we have extensive experience of transmission of a wide range of human acquired, sporadic and inherited prion disease isolates, including kuru and multiple vCJD cases (Collinge *et al.*, 1995a, b, 1996; Hill *et al.*, 1997; Wadsworth *et al.*, 2008a). Extensive comparative data are available on transmission of multiple cattle BSE isolates (Hill *et al.*, 1997; Asante *et al.*, 2002, 2006; Wadsworth *et al.*, 2004) as well as BSE experimentally passaged or naturally transmitted to multiple mammalian species and these mice are therefore suitable for comparative assessment of the zoonotic potential of CWD prions.

## RESULTS

### Immunoblot analysis of CWD-infected brain and spinal cord

**Fig. 1.**
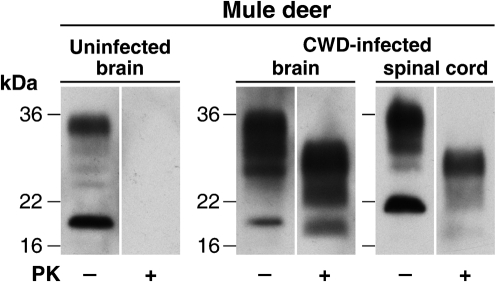
Detection of PrP^Sc^ in the brain and spinal cord from CWD-infected mule deer. Immunoblots show the analysis of 5 μl aliquots of 10 % (w/v) homogenates of uninfected mule deer brain or CWD-infected mule deer brain or spinal cord, before (−) or after (+) digestion with PK. Immunoblots were analysed by enhanced chemiluminescence with anti-PrP monoclonal antibody ICSM35.

CWD-infected mule deer brain (from animal D10) and spinal cord (from animal D08) originated from captive animals housed at the Colorado Division of Wildlife, Wildlife Research Centre, Colorado, USA. Homogenates (10 % w/v) of these tissues were prepared in PBS and examined for proteinase K (PK)-resistant PrP by immunoblotting. Both samples showed a high level of cervid PrP^Sc^ (Fig. 1) with a PrP glycoform ratio that showed a dominant diglycosylated conformer, typical of that associated with CWD prions (Race *et al.*, 2002). In contrast, identical analysis of brain homogenates prepared from uninfected mule deer showed no detectable PK-resistant PrP (Fig. 1 and data not shown).

### CWD prions do not transmit prion disease to transgenic mice overexpressing human prion protein

**Fig. 2.**
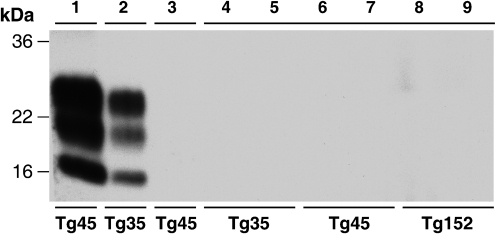
Failure to detect PrP^Sc^ in the brain of CWD prion-inoculated transgenic mice. The high sensitivity immunoblot using anti-PrP monoclonal antibody 3F4 shows PK-digested sodium phosphotungstic acid pellets recovered from 10 % (w/v) transgenic mouse brain homogenates. Lanes 1 and 2, positive controls showing efficient recovery of PrP^Sc^ after spiking 2 μl 10 % (w/v) BSE-inoculated 129MM Tg45 and 129MM Tg35 transgenic mouse brain homogenates (Asante *et al.*, 2002) into 100 μl 10 % (w/v) uninfected 129MM Tg45 and 129MM Tg35 mouse brain homogenates, respectively. Lane 3, PK-digested sodium phosphotungstic acid pellet from 250 μl 10 % (w/v) brain homogenate from a 129MM Tg45 mouse inoculated with normal mule deer brain. Lanes 4–9, PK-digested sodium phosphotungstic acid pellets from 250 μl 10 % (w/v) brain homogenates from 129MM Tg35, 129MM Tg45 and 129VV Tg152 mice inoculated with CWD-infected mule deer brain.

**Fig. 3.**
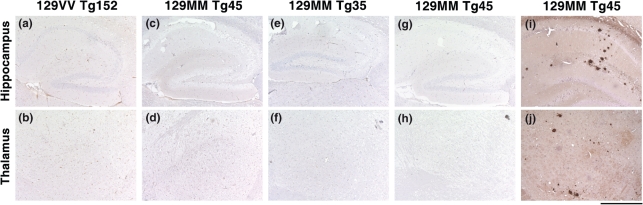
Failure to detect abnormal PrP deposition in the brain of CWD prion-inoculated transgenic mice. Representative PrP immunohistochemistry using anti-PrP monoclonal antibody ICSM35. Panels (a–f) show no abnormal PrP deposition in either the hippocampus or thalamus of 129VV Tg152, 129MM Tg45 or 129MM Tg35 mice inoculated with CWD-infected brain homogenate. These mice were culled 517, 529 and 559 days post-inoculation, respectively. Panels (g) and (h) show hippocampus and thalamus from an age matched control 129MM Tg45 mouse brain inoculated with 10 % (w/v) uninfected mule deer brain homogenate. In contrast, extensive deposition of abnormal PrP is seen in the hippocampus and thalamus of a BSE-infected 129MM Tg45 mouse with subclinical prion disease (panels i and j) (Asante *et al.*, 2002). Bar, 500 μm.

PrP^Sc^-positive CWD-infected brain and spinal cord homogenates were used to prepare inocula for transmission studies in transgenic mice overexpressing human PrP with either methionine or valine at polymorphic residue 129. 129MM Tg35, 129MM Tg45 and 129VV Tg152 transgenic mice overexpress human PrP in brain at levels of two, four and six times that of human brain, respectively (Collinge *et al.*, 1995b, 1996; Hill *et al.*, 1997; Asante *et al.*, 2002). These lines of mice have been extensively used by us for over 15 years and have proven susceptibility to infection with human or BSE prions (Collinge *et al.*, 1995b, 1996; Hill *et al.*, 1997; Asante *et al.*, 2002, 2006; Wadsworth *et al.*, 2004, 2007, 2008a). Following intracerebral inoculation with CWD brain or spinal cord, groups of 10 transgenic mice were observed throughout their life time for clinical signs of prion disease. As reported in Table 1, we observed no clinical prion disease in any inoculated mouse, including those with post-inoculation intervals greater than 700 days (Table 1). Accordingly, brains from mice culled as a result of inter-current illness or senescence were examined for subclinical prion transmission. In all cases examined, pathological PrP accumulation in brain was undetectable by either immunoblotting (Fig. 2, Table 1) or immunohistochemistry (Fig. 3, Table 1). Futhermore, neuropathological examination of CWD-inoculated transgenic mouse brain, showed no evidence of spongiform change or gliosis consistent with prion disease and their appearance was indistinguishable from the brain of age matched control mice inoculated with normal mule deer brain (Fig. 3 and data not shown). In summary, we conclude that intracerebral challenge of these transgenic mice with CWD prions caused no clinical or subclinical prion infection, indicating that both methionine and valine 129 polymorphs of human PrP are refractory to pathological conversion by CWD prions.

## DISCUSSION

In this study, we have shown that transgenic mice overexpressing human PrP of both residue 129 polymorphic forms, known to be susceptible to a wide range of human and other prions, are highly resistant to infection with mule deer CWD prions. These findings agree with those of others who have previously reported an inability of CWD prions to transmit disease to transgenic mice expressing human PrP 129 methionine (Kong *et al.*, 2005; Tamguney *et al.*, 2006) or a poor ability of human PrP to act as a substrate for CWD prions in *in vitro* conversion assays (Raymond *et al.*, 2000; Kurt *et al.*, 2009). Importantly, the transgenic mice used in our study have proven susceptibility to infection with BSE prions [Hill *et al.*, 1997; Asante *et al.*, 2002, 2006; Wadsworth *et al.*, 2004 (Table 1)]. The negative transmissions that we report here therefore strongly support the conclusion that the transmission barrier associated with the interaction of human PrP and these CWD prions is greater than that associated with interaction of human PrP and the prion strain causing epizootic BSE in cattle.

The failure to show propagation of CWD prions using human PrP as a substrate either *in vivo* in transgenic mice or *in vitro* in biochemical conversion assays suggests that potential zoonotic threat from CWD is low. However, an important caveat in this regard is that the number of prion strains propagated in CWD is currently unknown (Browning *et al.*, 2004; Raymond *et al.*, 2007; Green *et al.*, 2008; Angers *et al.*, 2010). Because prion strains can adapt and mutate on passage in new species (Collinge & Clarke, 2007; Beringue *et al.*, 2008; Castilla *et al.*, 2008; Collinge, 2010), and also within species as a result of PrP polymorphisms and other genetic factors (Asante *et al.*, 2002; Lloyd *et al.*, 2004; Wadsworth *et al.*, 2004; Mead *et al.*, 2009; Lloyd *et al.*, 2009), the risk that each prion strain poses to public health must be evaluated directly. There is now growing evidence that polymorphisms of cervid PrP may dictate prion strain selection (O'Rourke *et al.*, 2004; Meade-White *et al.*, 2007; Green *et al.*, 2008; Angers *et al.*, 2010). Thus, while the available experimental data appear reassuring, further transmission studies will be of vital importance to evaluate the properties of distinct cervid prion strains as they are isolated.

## METHODS

### Mule deer tissues.

Importation, storage and use of CWD-infected tissues was performed under licence granted by Defra under the terms of the Importation of Animal Pathogens Order 1980. CWD-infected mule deer brain (from animal D10) and spinal cord (from animal D08) originated from naturally infected captive animals from Colorado, USA that had clinical signs consistent with terminal stages of prion disease. CWD-infection in these animals was confirmed by the presence of histopathological lesions in the brain, including spongiform degeneration of the perikaryon, by immunohistochemical or immunoblot detection of disease-related PrP and by positive transmission of prion disease to transgenic mice expressing cervid PrP (Browning *et al.*, 2004; Angers *et al.*, 2006; Green *et al.*, 2008). Brain from uninfected mule deer fawns (FPS 6.98 and FPS 3.98) was used as negative controls.

### Transgenic mice.

Transgenic mice homozygous for a human PrP 129V transgene array and murine PrP null alleles (*Prnp^o/o^*) designated Tg(HuPrP129V^+/+^ *Prnp^o/o^*)-152 mice (129VV Tg152 mice) or homozygous for a human PrP 129M transgene array and murine PrP null alleles (*Prnp^o/o^*) designated Tg(HuPrP129M^+/+^ *Prnp^o/o^*)-35 mice (129MM Tg35 mice) or Tg(HuPrP129M^+/+^ *Prnp^o/o^*)-45 mice (129MM Tg45 mice) have been described previously (Collinge *et al.*, 1995b, 1996; Hill *et al.*, 1997; Asante *et al.*, 2002, 2006; Wadsworth *et al.*, 2004, 2007, 2008a).

### Transmission studies.

All procedures were carried out in a microbiological containment level 3 facility with strict adherence to safety protocols. Care of mice was according to institutional guidelines. Mule deer tissues were prepared as 10 % (w/v) homogenates in sterile PBS lacking Ca^2+^ and Mg^2+^ ions by serial passage through needles of decreasing diameter, and subsequently diluted to 1 % (w/v) in PBS. Following intracerebral inoculation with 30 μl of 1 % (w/v) tissue homogenate as described previously (Asante *et al.*, 2002, 2006; Wadsworth *et al.*, 2004), mice were examined daily and were killed if exhibiting signs of distress or once a diagnosis of clinical prion disease was established. Brains from inoculated mice were analysed by PrP immunoblotting or immunohistochemistry and by neuropathological examination.

### Immunoblotting.

All procedures were carried out in a microbiological containment level 3 facility with strict adherence to safety protocols. Tissue homogenates (10 % w/v) were prepared in PBS lacking Ca^2+^ or Mg^2+^ ions. PK digestion (50 or 100 μg ml^−1^ final protease concentration, 1 h, 37 °C), electrophoresis and immunoblotting was performed as described previously (Wadsworth *et al.*, 2001, 2008b). Immunoblot detection was performed using anti-PrP monoclonal antibody ICSM35 (D-Gen) for cervid PrP or 3F4 (Kascsak *et al.*, 1987) for human PrP in transgenic mice. Brain homogenates scored negative for PrP^Sc^ after analysis of 10 μl 10 % (w/v) brain homogenate were re-analysed by sodium phosphotungstic acid precipitation of PrP^Sc^ (Safar *et al.*, 1998) from 250 μl of 10 % (w/v) brain homogenate as described previously (Wadsworth *et al.*, 2001).

### Neuropathology and immunohistochemistry.

All steps prior to prion decontamination with formic acid were performed within a microbiological containment level 3 facility with strict adherence to safety protocols. Brain was fixed in 10 % buffered formal saline and then immersed in 98 % formic acid for 1 h and paraffin wax embedded. Serial sections of 4 μm thickness were pre-treated by boiling for 10 min in a low ionic strength buffer (2.1 mM Tris, 1.3 mM EDTA, 1.1 mM sodium citrate, pH 7.8) before exposure to 98 % formic acid for 5 min. Abnormal PrP accumulation was examined using anti-PrP monoclonal antibody ICSM35 (D-Gen) on a Ventana automated immunohistochemical staining machine (Ventana Medical Systems) using proprietary secondary detection reagents (Ventana Medical Systems) before development with 3′3-diaminobenzedine tetrachloride as the chromogen (Wadsworth *et al.*, 2008b). Harris haematoxylin and eosin staining was done by conventional methods. Appropriate positive and negative controls were used throughout. Photographs were taken on an ImageView digital camera and composed with Adobe Photoshop.

## Figures and Tables

**Table 1.** Primary transmission of CWD and BSE prions to transgenic mice Data for BSE transmissions have been published previously (Hill *et al.*, 1997; Asante *et al.*, 2002).

**Transgenic line**	**CWD brain**	**CWD spinal cord**	**BSE brain**
	Attack rate*	Attack rate*	Attack rate*
129VV Tg152	0/8†	0/6‡	10/26
129MM Tg45	0/7§	0/6||	9/12
129MM Tg35	0/9¶	0/5#	14/49

*All mice were inoculated with 30 μl of 1 % (w/v) tissue homogenate. Attack rate is defined as the total number of both clinically affected and subclinically infected mice as a proportion of the number of inoculated mice. Subclinical prion infection was assessed by sodium phosphotungstic acid precipitation of 250 μl 10 % brain homogenate and analysis for PrP^Sc^ by immunoblotting and/or immunohistochemical examination of brain. †Mice culled at 274, 316, 321, 436, 517, 517, 587 and 781 days post-inoculation. ‡Mice culled at 354, 364, 463, 541, 704 and 724 days post-inoculation. §Mice culled at 322, 322, 395, 400, 529, 656 and 736 days post-inoculation. ||Mice culled at 275, 345, 396, 462, 462 and 532 days post-inoculation. ¶Mice culled at 341, 559, 662, 662, 680, 707, 707, 747 and 748 days post-inoculation. #Mice culled at 392, 414, 542, 699 and 732 days post-inoculation.

## References

[r1] Anderson, C. A., Bosque, P., Filley, C. M., Arciniegas, D. B., Kleinschmidt-Demasters, B. K., Pape, W. J. & Tyler, K. L. (2007). Colorado surveillance program for chronic wasting disease transmission to humans: lessons from 2 highly suspicious but negative cases. Arch Neurol 64, 439–441. 17353391 10.1001/archneur.64.3.439

[r2] Angers, R. C., Browning, S. R., Seward, T. S., Sigurdson, C. J., Miller, M. W., Hoover, E. A. & Telling, G. C. (2006). Prions in skeletal muscles of deer with chronic wasting disease. Science 311, 1117. 16439622 10.1126/science.1122864

[r3] Angers, R. C., Kang, H. E., Napier, D., Browning, S., Seward, T., Mathiason, C., Balachandran, A., McKenzie, D., Castilla, J. & other authors (2010). Prion strain mutation determined by prion protein conformational compatibility and primary structure. Science 328, 1154–1158. 20466881 10.1126/science.1187107 PMC4097672

[r4] Asante, E. A., Linehan, J. M., Desbruslais, M., Joiner, S., Gowland, I., Wood, A. L., Welch, J., Hill, A. F., Lloyd, S. E. & other authors (2002). BSE prions propagate as either variant CJD-like or sporadic CJD-like prion strains in transgenic mice expressing human prion protein. EMBO J 21, 6358–6366. 12456643 10.1093/emboj/cdf653 PMC136957

[r5] Asante, E. A., Linehan, J. M., Gowland, I., Joiner, S., Fox, K., Cooper, S., Osiguwa, O., Gorry, M., Welch, J. & other authors (2006). Dissociation of pathological and molecular phenotype of variant Creutzfeldt–Jakob disease in transgenic human prion protein 129 heterozygous mice. Proc Natl Acad Sci U S A 103, 10759–10764. 16809423 10.1073/pnas.0604292103 PMC1502304

[r6] Baeten, L. A., Powers, B. E., Jewell, J. E., Spraker, T. R. & Miller, M. W. (2007). A natural case of chronic wasting disease in a free-ranging moose (*Alces alces shirasi*). J Wildl Dis 43, 309–314. 17495319 10.7589/0090-3558-43.2.309

[r7] Belay, E. D. (2004). Chronic wasting disease and potential transmission to humans. Emerg Infect Dis 10, 977–984. 15207045 10.3201/eid1006.031082 PMC3323184

[r8] Belay, E. D., Gambetti, P., Schonberger, L. B., Parchi, P., Lyon, D. R., Capellari, S., McQuiston, J. H., Bradley, K., Dowdle, G. & other authors (2001). Creutzfeldt–Jakob disease in unusually young patients who consumed venison. Arch Neurol 58, 1673–1678. 11594928 10.1001/archneur.58.10.1673

[r9] Beringue, V., Vilotte, J. L. & Laude, H. (2008). Prion agents diversity and species barrier. Vet Res 39, 47. 18519020 10.1051/vetres:2008024

[r10] Browning, S. R., Mason, G. L., Seward, T., Green, M., Eliason, G. A., Mathiason, C., Miller, M. W., Williams, E. S., Hoover, E. & other authors (2004). Transmission of prions from mule deer and elk with chronic wasting disease to transgenic mice expressing cervid PrP. J Virol 78, 13345–13350. 15542685 10.1128/JVI.78.23.13345-13350.2004 PMC524991

[r11] Bruce, M. E., Will, R. G., Ironside, J. W., McConnell, I., Drummond, D., Suttie, A., McCardle, L., Chree, A., Hope, J. & other authors (1997). Transmissions to mice indicate that ‘new variant’ CJD is caused by the BSE agent. Nature 389, 498–501. 9333239 10.1038/39057

[r12] Castilla, J., Gonzalez-Romero, D., Saa, P., Morales, R., De Castro, J. & Soto, C. (2008). Crossing the species barrier by PrP^Sc^ replication *in vitro* generates unique infectious prions. Cell 134, 757–768. 18775309 10.1016/j.cell.2008.07.030 PMC2740631

[r13] Collinge, J. (1999). Variant Creutzfeldt–Jakob disease. Lancet 354, 317–323. 10440324 10.1016/S0140-6736(99)05128-4

[r14] Collinge, J. (2010). Prion strain mutation and selection. Science 328, 1111–1112. 20508117 10.1126/science.1190815

[r15] Collinge, J. & Clarke, A. R. (2007). A general model of prion strains and their pathogenicity. Science 318, 930–936. 17991853 10.1126/science.1138718

[r16] Collinge, J., Palmer, M. S., Sidle, K. C. L., Gowland, I., Medori, R., Ironside, J. & Lantos, P. L. (1995a). Transmission of fatal familial insomnia to laboratory animals. Lancet 346, 569–570. 7658786 10.1016/s0140-6736(95)91405-6

[r17] Collinge, J., Palmer, M. S., Sidle, K. C. L., Hill, A. F., Gowland, I., Meads, J., Asante, E., Bradley, R., Doey, L. J. & other authors (1995b). Unaltered susceptibility to BSE in transgenic mice expressing human prion protein. Nature 378, 779–783. 8524411 10.1038/378779a0

[r18] Collinge, J., Sidle, K. C. L., Meads, J., Ironside, J. & Hill, A. F. (1996). Molecular analysis of prion strain variation and the aetiology of ‘new variant’ CJD. Nature 383, 685–690. 8878476 10.1038/383685a0

[r19] Collinge, J., Whitfield, J., McKintosh, E., Beck, J., Mead, S., Thomas, D. J. & Alpers, M. P. (2006). Kuru in the 21st century – an acquired human prion disease with very long incubation periods. Lancet 367, 2068–2074. 16798390 10.1016/S0140-6736(06)68930-7

[r20] Collinge, J., Whitfield, J., McKintosh, E., Frosh, A., Mead, S., Hill, A. F., Brandner, S., Thomas, D. & Alpers, M. P. (2008). A clinical study of kuru patients with long incubation periods at the end of the epidemic in Papua New Guinea. Philos Trans R Soc Lond B Biol Sci 363, 3725–3739. 18849289 10.1098/rstb.2008.0068 PMC2581654

[r21] Fox, K. A., Jewell, J. E., Williams, E. S. & Miller, M. W. (2006). Patterns of PrP^CWD^ accumulation during the course of chronic wasting disease infection in orally inoculated mule deer (*Odocoileus hemionus*). J Gen Virol 87, 3451–3461. 17030882 10.1099/vir.0.81999-0

[r22] Green, K. M., Browning, S. R., Seward, T. S., Jewell, J. E., Ross, D. L., Green, M. A., Williams, E. S., Hoover, E. A. & Telling, G. C. (2008). The elk PRNP codon 132 polymorphism controls cervid and scrapie prion propagation. J Gen Virol 89, 598–608. 18198392 10.1099/vir.0.83168-0

[r23] Guiroy, D. C., Williams, E. S., Song, K.-J., Yanagihara, R. & Gajdusek, D. C. (1993). Fibrils in brains of Rocky Mountain elk with chronic wasting disease contain scrapie amyloid. Acta Neuropathol 86, 77–80. 8372644 10.1007/BF00454902

[r24] Haley, N. J., Seelig, D. M., Zabel, M. D., Telling, G. C. & Hoover, E. A. (2009). Detection of CWD prions in urine and saliva of deer by transgenic mouse bioassay. PLoS ONE 4, e4848. 19293928 10.1371/journal.pone.0004848 PMC2654070

[r25] Hamir, A. N., Gidlewski, T., Spraker, T. R., Miller, J. M., Creekmore, L., Crocheck, M., Cline, T. & O'Rourke, K. I. (2006). Preliminary observations of genetic susceptibility of elk (*Cervus elaphus nelsoni*) to chronic wasting disease by experimental oral inoculation. J Vet Diagn Invest 18, 110–114. 16566268 10.1177/104063870601800118

[r26] Heisey, D. M., Mickelsen, N. A., Schneider, J. R., Johnson, C. J., Johnson, C. J., Langenberg, J. A., Bochsler, P. N., Keane, D. P. & Barr, D. J. (2010). Chronic wasting disease (CWD) susceptibility of several North American rodents that are sympatric with cervid CWD epidemics. J Virol 84, 210–215. 19828611 10.1128/JVI.00560-09 PMC2798418

[r27] Hill, A. F., Desbruslais, M., Joiner, S., Sidle, K. C. L., Gowland, I. & Collinge, J. (1997). The same prion strain causes vCJD and BSE. Nature 389, 448–450. 9333232 10.1038/38925

[r28] Jennelle, C. S., Samuel, M. D., Nolden, C. A., Keane, D. P., Barr, D. J., Johnson, C., Vanderloo, J. P., Aiken, J. M., Hamir, A. N. & other authors (2009). Surveillance for transmissible spongiform encephalopathy in scavengers of white-tailed deer carcasses in the chronic wasting disease area of Wisconsin. J Toxicol Environ Health A 72, 1018–1024. 19697235 10.1080/15287390903084249

[r29] Kascsak, R. J., Rubenstein, R., Merz, P. A., Tonna DeMasi, M., Fersko, R., Carp, R. I., Wisniewski, H. M. & Diringer, H. (1987). Mouse polyclonal and monoclonal antibody to scrapie-associated fibril proteins. J Virol 61, 3688–3693. 2446004 10.1128/jvi.61.12.3688-3693.1987 PMC255980

[r30] Kim, T. Y., Shon, H. J., Joo, Y. S., Mun, U. K., Kang, K. S. & Lee, Y. S. (2005). Additional cases of chronic wasting disease in imported deer in Korea. J Vet Med Sci 67, 753–759. 16141661 10.1292/jvms.67.753

[r31] Kong, Q., Huang, S., Zou, W., Vanegas, D., Wang, M., Wu, D., Yuan, J., Zheng, M., Bai, H. & other authors (2005). Chronic wasting disease of elk: transmissibility to humans examined by transgenic mouse models. J Neurosci 25, 7944–7949. 16135751 10.1523/JNEUROSCI.2467-05.2005 PMC6725448

[r32] Kong, Q., Zheng, M., Casalone, C., Qing, L., Huang, S., Chakraborty, B., Wang, P., Chen, F., Cali, I. & other authors (2008). Evaluation of the human transmission risk of an atypical bovine spongiform encephalopathy prion strain. J Virol 82, 3697–3701. 18234793 10.1128/JVI.02561-07 PMC2268471

[r33] Kurt, T. D., Telling, G. C., Zabel, M. D. & Hoover, E. A. (2009). Trans-species amplification of PrP^CWD^ and correlation with rigid loop 170N. Virology 387, 235–243. 19269662 10.1016/j.virol.2009.02.025

[r34] Lloyd, S. E., Linehan, J. M., Desbruslais, M., Joiner, S., Buckell, J., Brandner, S., Wadsworth, J. D. & Collinge, J. (2004). Characterization of two distinct prion strains derived from bovine spongiform encephalopathy transmissions to inbred mice. J Gen Virol 85, 2471–2478. 15269389 10.1099/vir.0.79889-0

[r35] Lloyd, S. E., Maytham, E. G., Pota, H., Grizenkova, J., Molou, E., Uphill, J., Hummerich, H., Whitfield, J., Alpers, M. P. & other authors (2009). HECTD2 is associated with susceptibility to mouse and human prion disease. PLoS Genet 5, e1000383. 19214206 10.1371/journal.pgen.1000383 PMC2633041

[r36] Marsh, R. F., Kincaid, A. E., Bessen, R. A. & Bartz, J. C. (2005). Interspecies transmission of chronic wasting disease prions to squirrel monkeys (*Saimiri sciureus*). J Virol 79, 13794–13796. 16227298 10.1128/JVI.79.21.13794-13796.2005 PMC1262585

[r37] Mathiason, C. K., Powers, J. G., Dahmes, S. J., Osborn, D. A., Miller, K. V., Warren, R. J., Mason, G. L., Hays, S. A., Hayes-Klug, J. & other authors (2006). Infectious prions in the saliva and blood of deer with chronic wasting disease. Science 314, 133–136. 17023660 10.1126/science.1132661

[r38] Mathiason, C. K., Hays, S. A., Powers, J., Hayes-Klug, J., Langenberg, J., Dahmes, S. J., Osborn, D. A., Miller, K. V., Warren, R. J. & other authors (2009). Infectious prions in pre-clinical deer and transmission of chronic wasting disease solely by environmental exposure. PLoS ONE 4, e5916. 19529769 10.1371/journal.pone.0005916 PMC2691594

[r39] Mawhinney, S., Pape, W. J., Forster, J. E., Anderson, C. A., Bosque, P. & Miller, M. W. (2006). Human prion disease and relative risk associated with chronic wasting disease. Emerg Infect Dis 12, 1527–1535. 17176567 10.3201/eid1210.060019 PMC3290936

[r40] Mead, S., Poulter, M., Uphill, J., Beck, J., Whitfield, J., Webb, T. E., Campbell, T., Adamson, G., Deriziotis, P. & other authors (2009). Genetic risk factors for variant Creutzfeldt–Jakob disease: a genome-wide association study. Lancet Neurol 8, 57–66. 19081515 10.1016/S1474-4422(08)70265-5 PMC2643048

[r41] Meade-White, K., Race, B., Trifilo, M., Bossers, A., Favara, C., LaCasse, R., Miller, M., Williams, E., Oldstone, M. & other authors (2007). Resistance to chronic wasting disease (CWD) in transgenic mice expressing a naturally occurring allelic variant of deer prion protein. J Virol 81, 4533–4539. 17314157 10.1128/JVI.02762-06 PMC1900179

[r42] Nichols, T. A., Pulford, B., Wyckoff, A. C., Meyerett, C., Michel, B., Gertig, K., Hoover, E. A., Jewell, J. E., Telling, G. C. & other authors (2009). Detection of protease-resistant cervid prion protein in water from a CWD-endemic area. Prion 3, 171–183. 19823039 10.4161/pri.3.3.9819 PMC2802782

[r43] O'Rourke, K. I., Spraker, T. R., Hamburg, L. K., Besser, T. E., Brayton, K. A. & Knowles, D. P. (2004). Polymorphisms in the prion precursor functional gene but not the pseudogene are associated with susceptibility to chronic wasting disease in white-tailed deer. J Gen Virol 85, 1339–1346. 15105552 10.1099/vir.0.79785-0

[r44] Prusiner, S. B. (1998). Prions. Proc Natl Acad Sci U S A 95, 13363–13383. 9811807 10.1073/pnas.95.23.13363 PMC33918

[r45] Race, R. E., Raines, A., Baron, T. G., Miller, M. W., Jenny, A. & Williams, E. S. (2002). Comparison of abnormal prion protein glycoform patterns from transmissible spongiform encephalopathy agent-infected deer, elk, sheep, and cattle. J Virol 76, 12365–12368. 12414979 10.1128/JVI.76.23.12365-12368.2002 PMC136873

[r46] Race, B., Meade-White, K., Race, R. & Chesebro, B. (2009a). Prion infectivity in fat of deer with chronic wasting disease. J Virol 83, 9608–9610. 19570855 10.1128/JVI.01127-09 PMC2738259

[r47] Race, B., Meade-White, K. D., Miller, M. W., Barbian, K. D., Rubenstein, R., LaFauci, G., Cervenakova, L., Favara, C., Gardner, D. & other authors (2009b). Susceptibilities of nonhuman primates to chronic wasting disease. Emerg Infect Dis 15, 1366–1376. 19788803 10.3201/eid1509.090253 PMC2819871

[r48] Raymond, G. J., Bossers, A., Raymond, L. D., O'Rourke, K. I., McHolland, L. E., Bryant, P. K., III, Miller, M. W., Williams, E. S., Smits, M. & other authors (2000). Evidence of a molecular barrier limiting susceptibility of humans, cattle and sheep to chronic wasting disease. EMBO J 19, 4425–4430. 10970836 10.1093/emboj/19.17.4425 PMC302048

[r49] Raymond, G. J., Raymond, L. D., Meade-White, K. D., Hughson, A. G., Favara, C., Gardner, D., Williams, E. S., Miller, M. W., Race, R. E. & other authors (2007). Transmission and adaptation of chronic wasting disease to hamsters and transgenic mice: evidence for strains. J Virol 81, 4305–4314. 17287284 10.1128/JVI.02474-06 PMC1866158

[r50] Safar, J., Wille, H., Itri, V., Groth, D., Serban, H., Torchia, M., Cohen, F. E. & Prusiner, S. B. (1998). Eight prion strains have PrP^Sc^ molecules with different conformations. Nat Med 4, 1157–1165. 9771749 10.1038/2654

[r51] Sigurdson, C. J. (2008). A prion disease of cervids: chronic wasting disease. Vet Res 39, 41. 18381058 10.1051/vetres:2008018

[r52] Sigurdson, C. J. & Aguzzi, A. (2007). Chronic wasting disease. Biochim Biophys Acta 1772, 610–618. 17223321 10.1016/j.bbadis.2006.10.010 PMC2680674

[r53] Sigurdson, C. J., Mathiason, C. K., Perrott, M. R., Eliason, G. A., Spraker, T. R., Glatzel, M., Manco, G., Bartz, J. C., Miller, M. W. & other authors (2008). Experimental chronic wasting disease (CWD) in the ferret. J Comp Pathol 138, 189–196. 18387626 10.1016/j.jcpa.2008.01.004

[r54] Tamguney, G., Giles, K., Bouzamondo-Bernstein, E., Bosque, P. J., Miller, M. W., Safar, J., DeArmond, S. J. & Prusiner, S. B. (2006). Transmission of elk and deer prions to transgenic mice. J Virol 80, 9104–9114. 16940522 10.1128/JVI.00098-06 PMC1563923

[r55] Tamguney, G., Miller, M. W., Wolfe, L. L., Sirochman, T. M., Glidden, D. V., Palmer, C., Lemus, A., DeArmond, S. J. & Prusiner, S. B. (2009). Asymptomatic deer excrete infectious prions in faeces. Nature 461, 529–532. 19741608 10.1038/nature08289 PMC3186440

[r56] Trifilo, M. J., Ying, G., Teng, C. & Oldstone, M. B. (2007). Chronic wasting disease of deer and elk in transgenic mice: oral transmission and pathobiology. Virology 365, 136–143. 17451773 10.1016/j.virol.2007.03.032 PMC1950321

[r57] Wadsworth, J. D. & Collinge, J. (2007). Update on human prion disease. Biochim Biophys Acta 1772, 598–609. 17408929 10.1016/j.bbadis.2007.02.010

[r58] Wadsworth, J. D., Joiner, S., Hill, A. F., Campbell, T. A., Desbruslais, M., Luthert, P. J. & Collinge, J. (2001). Tissue distribution of protease resistant prion protein in variant CJD using a highly sensitive immuno-blotting assay. Lancet 358, 171–180. 11476832 10.1016/s0140-6736(01)05403-4

[r59] Wadsworth, J. D., Asante, E., Desbruslais, M., Linehan, J., Joiner, S., Gowland, I., Welch, J., Stone, L., Lloyd, S. & other authors (2004). Human prion protein with valine 129 prevents expression of variant CJD phenotype. Science 306, 1793–1796. 15539564 10.1126/science.1103932

[r60] Wadsworth, J. D., Joiner, S., Fox, K., Linehan, J., Desbruslais, M., Brandner, S., Asante, E. & Collinge, J. (2007). Prion infectivity in variant Creutzfeldt–Jakob disease rectum. Gut 56, 90–94. 16763054 10.1136/gut.2006.091637 PMC1856674

[r61] Wadsworth, J. D., Joiner, S., Linehan, J. M., Desbruslais, M., Fox, K., Cooper, S., Cronier, S., Asante, E. A., Mead, S. & other authors (2008a). Kuru prions and sporadic Creutzfeldt–Jakob disease prions have equivalent transmission properties in transgenic and wild-type mice. Proc Natl Acad Sci U S A 105, 3885–3890. 18316717 10.1073/pnas.0800190105 PMC2268835

[r62] Wadsworth, J. D., Powell, C., Beck, J. A., Joiner, S., Linehan, J. M., Brandner, S., Mead, S. & Collinge, J. (2008b). Molecular diagnosis of human prion disease. Methods Mol Biol 459, 197–227. 18576157 10.1007/978-1-59745-234-2_14

[r63] Williams, E. S. (2005). Chronic wasting disease. Vet Pathol 42, 530–549. 16145200 10.1354/vp.42-5-530

[r64] Williams, E. S. & Young, S. (1980). Chronic wasting disease of captive mule deer: a spongiform encephalopathy. J Wildl Dis 16, 89–98. 7373730 10.7589/0090-3558-16.1.89

[r65] Williams, E. S. & Young, S. (1982). Spongiform encephalopathy of Rocky Mountain elk. J Wildl Dis 18, 465–471. 7154220 10.7589/0090-3558-18.4.465

